# Supplementary material for: FinaleMe: Predicting DNA methylation by the fragmentation patterns of plasma cell-free DNA
Source: Nat Commun. 2024 Mar 30;15:2790. doi: 10.1038/s41467-024-47196-6 (PMC10981715; doi:10.1038/s41467-024-47196-6)
Supplement: Supplementary file 3 — Description of Additional Supplementary Files [file 41467_2024_47196_MOESM3_ESM.pdf]

Description of Additional Supplementary Files

File Name: Supplementary Data 1
Description: Meta-data information for each sample.

File Name: Supplementary Data 2
Description: Batch information for cfDNA WGS data.

File Name: Supplementary Data 3
Description: Batch information for cfDNA WGBS data.
