# Supplementary material for: FinaleMe: Predicting DNA methylation by the fragmentation patterns of plasma cell-free DNA
Source: Nat Commun. 2024 Mar 30;15:2790. doi: 10.1038/s41467-024-47196-6 (PMC10981715; doi:10.1038/s41467-024-47196-6)
Supplement: Supplementary file 5 — Reporting Summary [file 41467_2024_47196_MOESM5_ESM.pdf]

nature portfolio

Corresponding author(s): *Double-anonymous peer review submissions: write DAPR and your manuscript number here instead of author names.*

Last updated by author(s): *YYYY-MM-DD*

Reporting Summary

Nature Portfolio wishes to improve the reproducibility of the work that we publish. This form provides structure for consistency and transparency in reporting. For further information on Nature Portfolio policies, see our Editorial Policies and the Editorial Policy Checklist.

Statistics

For all statistical analyses, confirm that the following items are present in the figure legend, table legend, main text, or Methods section.

n/a	Confirmed	
☐	☒	The exact sample size (*n*) for each experimental group/condition, given as a discrete number and unit of measurement
☐	☒	A statement on whether measurements were taken from distinct samples or whether the same sample was measured repeatedly
☐	☒	The statistical test(s) used AND whether they are one- or two-sided<br>*Only common tests should be described solely by name; describe more complex techniques in the Methods section.*
☒	☐	A description of all covariates tested
☒	☐	A description of any assumptions or corrections, such as tests of normality and adjustment for multiple comparisons
☒	☐	A full description of the statistical parameters including central tendency (e.g. means) or other basic estimates (e.g. regression coefficient) AND variation (e.g. standard deviation) or associated estimates of uncertainty (e.g. confidence intervals)
☐	☒	For null hypothesis testing, the test statistic (e.g. *F*, *t*, *r*) with confidence intervals, effect sizes, degrees of freedom and *P* value noted<br>*Give P values as exact values whenever suitable.*
☐	☒	For Bayesian analysis, information on the choice of priors and Markov chain Monte Carlo settings
☒	☐	For hierarchical and complex designs, identification of the appropriate level for tests and full reporting of outcomes
☒	☐	Estimates of effect sizes (e.g. Cohen's *d*, Pearson's *r*), indicating how they were calculated

Our web collection on statistics for biologists contains articles on many of the points above.

Software and code

Policy information about availability of computer code

Data collection	no software used for the data collection
Data analysis	For WGS data, reads were aligned to the human genome (GRCh37) using BWA-MEM 0.7.15 with default parameters.<br>For WGBS data, reads were aligned to the human genome (GRCh37) using Bismark (v0.22.3) with bowtie2 (v2.3.5)<br>). The methylation level from WGBS was called by Bis-SNP v0.90<br>Estimation of tumor fraction was performed using ichorCNA (v0.2.0) as described previously in Adalsteinsson et al. Nature Communications 2017<br>Code for FinaleMe and associated scripts are publicly available on GitHub under the MIT license for academic researchers: https://github.com/epifluidlab/FinaleMe.git.

For manuscripts utilizing custom algorithms or software that are central to the research but not yet described in published literature, software must be made available to editors and reviewers. We strongly encourage code deposition in a community repository (e.g. GitHub). See the Nature Portfolio guidelines for submitting code & software for further information.

Data

Policy information about availability of data

All manuscripts must include a data availability statement. This statement should provide the following information, where applicable:

- Accession codes, unique identifiers, or web links for publicly available datasets
- A description of any restrictions on data availability
- For clinical datasets or third party data, please ensure that the statement adheres to our policy

The publicly available cfDNA WGBS data used in this study are available in the dbGaP database under accession code [https://www.ncbi.nlm.nih.gov/projects/gap/cgi-bin/study.cgi?study_id=phs000846.v1.p1]7. The publicly available ULP-WGS data used in this study are available in the dbGaP database under accession code [https://www.ncbi.nlm.nih.gov/projects/gap/cgi-bin/study.cgi?study_id=phs001417.v1.p1]25. The raw sequencing data for the deep WGS, WGBS, and ULP-WGBS data generated in this study have been deposited in the Sequence Read Archive with controlled access from dbGaP under accession code phs003287.v1.p1 [https://www.ncbi.nlm.nih.gov/projects/gap/cgi-bin/study.cgi?study_id=phs003287.v1.p1]. These data are available under restricted access due to individual privacy concerns. Permanent employees of an institution at a level equivalent to a tenure-track professor or senior scientist with laboratory administration and oversight responsibilities may request access through dbGaP. The requests, which are managed by NHGRI's Data Access Committee, take less than one month for approval, and access is permitted for 12 months. The processed and de-identified data are available in zenodo.org (doi: https://doi.org/10.5281/zenodo.7779198)35. The remaining data are available within the Article, Supplementary Information, and Source Data file.

Research involving human participants, their data, or biological material

Policy information about studies with human participants or human data. See also policy information about sex, gender (identity/presentation), and sexual orientation and race, ethnicity and racism.

Reporting on sex and gender	the analysis were done for autosomes only. the sex and gender information was not collected
Reporting on race, ethnicity, or other socially relevant groupings	the race, ethnicity information was not collected since this is not relevant for the method development study here.
Population characteristics	see above
Recruitment	Samples are from previously collected retrospective cohort
Ethics oversight	This research study was approved by the Broad Institute Institutional Review Board in accordance with the Declaration of Helsinki. De-identified plasma sample collection was approved by the Dana-Farber Cancer Institute and Broad Institute Institutional Review Boards. All participants provided written informed consent to participate.

Note that full information on the approval of the study protocol must also be provided in the manuscript.

Field-specific reporting

Please select the one below that is the best fit for your research. If you are not sure, read the appropriate sections before making your selection.

☒ Life sciences ☐ Behavioural & social sciences ☐ Ecological, evolutionary & environmental sciences

For a reference copy of the document with all sections, see nature.com/documents/nr-reporting-summary-flat.pdf

Life sciences study design

All studies must disclose on these points even when the disclosure is negative.

Sample size	this is a computational method development paper. We just used all the samples available at hand
Data exclusions	no data exclusion
Replication	we have replicates for deep coverage and ultra-low pass samples. and all replicates have been done successfully.
Randomization	this is a computational method development paper. we randomly generated cfDNA libraries.
Blinding	this is a computational method development paper. we used de-identified samples and blinding is not relevant to this study.

Reporting for specific materials, systems and methods

We require information from authors about some types of materials, experimental systems and methods used in many studies. Here, indicate whether each material, system or method listed is relevant to your study. If you are not sure if a list item applies to your research, read the appropriate section before selecting a response.

Materials & experimental systems

n/a	Involved in the study
☒	☐ Antibodies
☒	☐ Eukaryotic cell lines
☒	☐ Palaeontology and archaeology
☒	☐ Animals and other organisms
☒	☐ Clinical data
☒	☐ Dual use research of concern
☒	☐ Plants

Methods

n/a	Involved in the study
☒	☐ ChIP-seq
☒	☐ Flow cytometry
☒	☐ MRI-based neuroimaging

Plants

Seed stocks: NA

Novel plant genotypes: NA

Authentication: NA
